# Supplementary material for: Anticipated stigma and associated factors among chronic illness patients in Amhara Region Referral Hospitals, Ethiopia: A multicenter cross-sectional study
Source: PLoS One. 2022 Sep 15;17(9):e0273734. doi: 10.1371/journal.pone.0273734 (PMC9477324; doi:10.1371/journal.pone.0273734)
Supplement: S1 Questionnaire — (DOCX) [file pone.0273734.s002.docx]

# Questionnaire for Anticipated stigma and associated factors among chronic illness patients in Amhara Region Referral Hospitals, Ethiopia English questionnaire

**CONSENT**

**Questions for tool validation Information sheet:**

Read the statements to the respondent

**Purpose of the research project**

Dear Participants,

The University of Gondar School of Nursing and principal investigator are conducting the study in three areas of Amhara region referral Hospitals, Ethiopia. Namely University of Gondar Comprehensive Specialized Referral Hospital, Felege-Hiwot Referral Hospitals, and Dessie Referral Hospital. After conducting the tool validation study we are plan to assess the level and associated factors of anticipated stigma among three referral hospitals. The overall aim of the study is to evaluate the level and associated factors among patients with chronic illness attending follow-up clinics in Amhara Region Referral Hospitals, Ethiopia. You have been chosen randomly to participate in this study.

**Procedure:** To collect our data, we invite you to take part in our project. If you are willing, you need to understand and sign the consent form. We will ask questions related to your chronic illness's anticipated stigma.

**Risk and/or discomfort/t:** By participating in this research project you may feel some discomfort especially on sacrificing your time, otherwise is no risk in participating in this study.

**Benefits:** If you are participating in this research project, the output of the study will have both direct and indirect benefits to you, as well as your family and the community at large will get better treatment and services in the future.

**Incentives/payments for participating:** You will not be provided any incentives or payments to take part in this project.

**Confidentiality:** The information collected from this research project will be kept confidential and information about you that will be collected by this study will be stored in a file, without your name, but a code number assigned to it. And it will not be revealed to anyone except the principal investigator and will be kept locked with a key.

**Right to refuse or withdraw:** You have the full right to refuse from participating in this research. Your refusal will not affect you from getting any kind of health-related service.

**Person to contact:** If you want to know more information you can contact;

Mr. Mohammed Hassen by Tel: mobile 251-918785735

IRB of UOG by Tel: 251-581141231

If you agree to participate in the study described above, please sign below/ put your finger signature and write the date. Please respond to the interviewer with the question in the order. Instructions for how to respond to the different questions will be provided by the interviewer.

I, the undersigned, have understood the objective of the project is “to evaluate the magnitude of anticipated stigma and associated factors among patients with chronic illness attending follow-up clinics in Amhara Region Referral Hospitals, Ethiopia. ”, and agreed to be included in the study as explained by the researchers. For this, I also agree to fill the questionnaire which will be interviewed by the interviewer.

Yes ________________ Signature (fingerprint)________________________

No______________Signature (fingerprint)_______________(Terminate the interview)

Date_________________Time started ____________and completed______________

Code Number_________ Area code_____________ Identifier:____________________

**Overall information of the participant**

| **Location and date** | | **Response** |
| --- | --- | --- |
| 1 | Interviewer ID | |
| 2 | Patient ID | |
| 3 | Institution ID | |
| 4 | Date of completion of the instrument (DD/MM/YR ) | |

**Instruction:- Please respond to the following question based on the choices you have**

**Part I. Sociodemographic and institutional characteristics of the study participant**

| **Code** | **Question** | **Response** | **Remarks** |
| --- | --- | --- | --- |
| 101 | What is your sex? | 1. Male 2. Female |  |
| 102 | What is your age | ____________Yrs |  |
| 103 | Residency | 1. Urban 2. Rural |  |
| 104 | Ethnicity | 1. Amhara 2. Oromo 3. Afar 4. Tigre 5. Other (Pls specify_______) |  |
| 105 | Religion | 1. Orthodox 2. Muslim 3. Catholic 4. Protestant 5. Other ( Pls specify ______) |  |
| 106 | Educational Status | 1. Not read and write  2. Read and write  3. primary (1-8 grade)  4. Secondary (9-10 grade)  5. preparatory (11-12 grade)  6. technic schools  7. tertiary (diploma and above) |  |
| 107 | Marital Status | \| 1. Single 2. married  3. Separated 4. divorced  5. widowed \| \| --- \| |  |
| 108 | Occupation | _____________________ |  |
| 109 | Monthly Income | ___________________ETB |  |
| 110 | Access to media related to health | 1. No 2. Yes | If No pls go to 13 |
| 111 | Type of media access (More than one response is possible) | 1. Tv 3. Newspaper/magazine  2. Radio |  |
| 112 | Fear of contagion in the hospital | 1. No 1. Yes |  |
| 113 | Attending regular health service | 1. No 1. Yes |  |

**Part II:- Clinical and personal Factors**

| **Code** | **Question** | **Responses** | **Remark** |
| --- | --- | --- | --- |
| 201 | Primary chronic disease diagnosis |  |  |
| 202 | Comorbidity | 1, No  2, Yes | If your response is 0 pls got to Q204 |
| 203 | How many comorbidities? | ____________________ |  |
| 204 | Duration of chronic illness | ____________yrs |  |
| 205 | Type of medication  (You can tick more than one response) | 1, Analgesics 2, Cardio-vascular  3, Respiratory 4, Endocrine  5, Chemotherapy 6, ART  7, Other (Pls list___________) |  |
| 206 | Are you a smoker | 1, Non- smokers   1. former smokers (ex-smokers) 2. Occasional Smoker 3. Daily smokers |  |
| 207 | If daily smoker (4) how many cigarette / day? | _____________ Cigarette |  |
| 208 | Have you ever tried chat chewing? | No  Yes | If No pls got to part III |
| 209 | Are you currently taking chat | 1. No 2. Yes |  |

**Part III. Simplified Medication Adherence questionnaire (SMAQ)**

| **S.no** | **Questions** | **Responses** | **Remark** |
| --- | --- | --- | --- |
| 301 | Do you ever forget to take your medicine? | 1. Yes 2. Never |  |
| 302 | Are you careless at times about taking your medicine? | 1. Yes 2. Never |  |
| 303 | Sometimes if you feel worse, do you stop taking your medicines? | 1. Yes 2. Never |  |
| 304 | Thinking about the last week. How often have you not taken your medicine | 1. Never  2. 1 + 2 times  3. 3 + 5 times   1. 6 + 10 times 2. More than 10 times |  |
| 305 | Did you not take any of your medicine over the past weekend? | - - - 1. Never       2. Yes |  |
| 306 | Over the past 3 months, how many days have you not taken any medicine at all? | 1. < 2 days 2. > 2 days |  |

**Part IV. The Chronic Illness Anticipated Stigma Scale (CIASS)**

**Instructions**: The statements listed below describe some of the ways that people who have chronic illnesses are treated by others. Please read these statements and mark how likely you think that they could happen to you in the future.

First, think about how your friends and family members such as parents, sisters and brothers, and children will treat you in the future. How likely is it that they will treat you in the following way. **Because of your chronic illness?**

| **S.no** | **Questions** | **Very**  **Unlikely** | **Unlikely** | **Somewhat likely** | **Likely** | **Very Likely** | **Remark** |
| --- | --- | --- | --- | --- | --- | --- | --- |
| 401 | A friend or family member will be angry with you. | 1 | 2 | 3 | 4 | 5 |  |
| 402 | A friend or family member will blame you for not getting better. | 1 | 2 | 3 | 4 | 5 |  |
| 403 | A friend or family member will think that your illness is your fault. | 1 | 2 | 3 | 4 | 5 |  |
| 404 | A friend or family member will not think as highly of you. | 1 | 2 | 3 | 4 | 5 |  |

Now, think about how your coworkers and employers will treat you in the future. If you are not currently employed, think about coworkers and employers that you might have in the future. How likely is it that they will treat you in the following ways **because of your chronic illness?**

| **S.no** | **Question** | **Very**  **Unlikely** | **Unlikely** | **Somewhat likely** | **Likely** | **Very Likely** | **Remark** |
| --- | --- | --- | --- | --- | --- | --- | --- |
| 405 | Your employer will not promote you. | 1 | 2 | 3 | 4 | 5 |  |
| 406 | Someone at work will discriminate against you. | 1 | 2 | 3 | 4 | 5 |  |
| 407 | Your employer will assign a challenging project to someone else. | 1 | 2 | 3 | 4 | 5 |  |
| 408 | Someone at work will think that you cannot fulfill your work responsibilities | 1 | 2 | 3 | 4 | 5 |  |

Finally, think about how healthcare providers such as doctors, nurses, technicians, and secretaries who work at hospitals and doctor’s offices will treat you in the future. How likely is it that they will treat you in the following ways **because of your chronic illness?**

| S.no |  | Very  Unlikely | Unlikely | Somewhat likely | Likely | Very Likely | Remark |
| --- | --- | --- | --- | --- | --- | --- | --- |
| 409 | A health care worker will be frustrated with you. | 1 | 2 | 3 | 4 | 5 |  |
| 410 | A health care worker will give you poor care. | 1 | 2 | 3 | 4 | 5 |  |
| 411 | A health care worker will blame you for not getting better. | 1 | 2 | 3 | 4 | 5 |  |
| 412 | A health care worker will think that you are a bad patient. | 1 | 2 | 3 | 4 | 5 |  |

**Part V. WHOQOL- BREF**

The following questions ask how you feel about your quality of life, health, or other areas of your life. I will read out each question to you, along with the response options. **Please choose the answer that appears most appropriate.** If you are unsure about which response to give to a question, the first response you think of is often the best one.

Please keep in mind your standards, hopes, pleasures, and concerns. We ask that you think about your life **in the last four weeks.**

| **S.No** | **Question** | | **Very poor** | **Poor** | | **Neither poor nor good** | | **Good** | **Very good** | **Remark** | |
| --- | --- | --- | --- | --- | --- | --- | --- | --- | --- | --- | --- |
| 501 | How would you rate your quality of life? | | 1 | 2 | | 3 | | 4 | 5 |  | |
| **S.No** | **Question** | **Very dissatisfied** | **Dissatisfied** | | **Neither satisfied nor dissatisfied** | | **Satisfied** | | **Very satisfied** | | **Remark** |
| 502 | How satisfied are you with your health? | 1 | 2 | | 3 | | 4 | | 5 | |  |

The following questions ask about **how much** you have experienced certain things in the **last four weeks.**

| **S.no** | **Question** | | **Not at all** | | **A little** | | **A moderate amount** | **Very much** | | **An extreme amount** | **Remark** |  |
| --- | --- | --- | --- | --- | --- | --- | --- | --- | --- | --- | --- | --- |
| 503 | To what extent do you feel that physical pain prevents you from doing what you need to do? | | 1 | | 2 | | 3 | 4 | | 5 |  |  |
| 504 | How much do you need any medical treatment to function in your daily life? | | 1 | | 2 | | 3 | 4 | | 5 |  |  |
| 505 | How much do you enjoy life? | | 1 | | 2 | | 3 | 4 | | 5 |  |  |
| 506 | To what extent do you feel your life to be meaningful? | | 1 | | 2 | | 3 | 4 | | 5 |  |  |
| 507 | How well are you able to concentrate? | | 1 | | 2 | | 3 | 4 | | 5 |  |  |
| **S.no** | **Question** | **Not at all** | | **A little** | | **A moderate amount** | | **Very much** | **Extremely** | | **Remark** | |
| 508 | How safe do you feel in your daily life? | 1 | | 2 | | 3 | | 4 | 5 | |  | |
| 509 | How healthy is your physical environment? | 1 | | 2 | | 3 | | 4 | 5 | |  | |

The following questions ask about how completely you experience or were able to do certain things in **the last four weeks.**

| **S.no** | **Question** | | | **Not at all** | | **A little** | **Moderately** | | **Mostly** | | **Completely** | | | **Remark** | | |
| --- | --- | --- | --- | --- | --- | --- | --- | --- | --- | --- | --- | --- | --- | --- | --- | --- |
| 510 | Do you have enough energy for everyday life? | | | 1 | | 2 | 3 | | 4 | | 5 | | |  | | |
| 511 | Are you able to accept your bodily appearance? | | | 1 | | 2 | 3 | | 4 | | 5 | | |  | | |
| 512 | Have you enough money to meet your needs? | | | 1 | | 2 | 3 | | 4 | | 5 | | |  | | |
| 513 | How available to you is the information that you need in your day-to-day life? | | | 1 | | 2 | 3 | | 4 | | 5 | | |  | | |
| 514 | To what extent do you have the opportunity for leisure activities? | | | 1 | | 2 | 3 | | 4 | | 5 | | |  | | |
| **S.NO** | **Question** | | **Very poor** | | **Poor** | | **Neither poor nor good** | | | **Good** | | **Very good** | | | **Remark** | |
| 515 | How well are you able to get around? | | 1 | | 2 | | 3 | | | 4 | | 5 | | |  | |
| **S.no** | **Questions** | **Very dissatisfied** | | | **Dissatisfied** | | | **Neither satisfied nor dissatisfied** | | **Satisfied** | | | **Very satisfied** | | | **Remark** |
| 516 | How satisfied are you with your sleep? | 1 | | | 2 | | | 3 | | 4 | | | 5 | | |  |
| 517 | How satisfied are you with your ability to perform your daily living activities? | 1 | | | 2 | | | 3 | | 4 | | | 5 | | |  |
| 518 | How satisfied are you with your capacity for work? | 1 | | | 2 | | | 3 | | 4 | | | 5 | | |  |
| 519 | How satisfied are you with yourself? | 1 | | | 2 | | | 3 | | 4 | | | 5 | | |  |
| 520 | How satisfied are you with your relationships? | 1 | | | 2 | | | 3 | | 4 | | | 5 | | |  |
| 521 | How satisfied are you with your sex life? | 1 | | | 2 | | | 3 | | 4 | | | 5 | | |  |
| 522 | How satisfied are you with the support you get from your friends? | 1 | | | 2 | | | 3 | | 4 | | | 5 | | |  |
| 523 | How satisfied are you with the conditions of your living place? | 1 | | | 2 | | | 3 | | 4 | | | 5 | | |  |
| 524 | How satisfied are you with your access to health services? | 1 | | | 2 | | | 3 | | 4 | | | 5 | | |  |
| 525 | How satisfied are you with your transport? | 1 | | | 2 | | | 3 | | 4 | | | 5 | | |  |

The following question refers to how often you have felt or experienced certain things in **the last four weeks**

| **S. No** | **Question** | **Never** | **Seldom** | **Quite often** | **Very often** | **Always** | **Remark** |
| --- | --- | --- | --- | --- | --- | --- | --- |
| 526 | How often do you have negative feelings such as blue mood, despair, anxiety, depression? | 1 | 2 | 3 | 4 | 5 |  |

**Part VI. Kessler psychological distress scale (CMD)**

These questions concern how you have been feeling over the **past 30 days**. Tick a box below each question that best represents how you have been.

| S.no | Question | None of the time | A little of the time | Some of the Time | Most of the time | All of the time | Remark |
| --- | --- | --- | --- | --- | --- | --- | --- |
| 601 | During the last 30 days, about how often did you feel tired out for no good reason? | 1 | 2 | 3 | 4 | 5 |  |
| 602 | During the last 30 days, about how often did you feel nervous? | 1 | 2 | 3 | 4 | 5 |  |
| 603 | During the last 30 days, about how often did you feel so nervous that nothing could calm you down? | 1 | 2 | 3 | 4 | 5 |  |
| 604 | During the last 30 days, about how often did you feel hopeless? | 1 | 2 | 3 | 4 | 5 |  |
| 605 | During the last 30 days, about how often did you feel restless or fidgety? | 1 | 2 | 3 | 4 | 5 |  |
| 606 | During the last 30 days, about how often did you feel so restless you could not sit still? | 1 | 2 | 3 | 4 | 5 |  |
| 607 | During the last 30 days, about how often did you feel depressed? | 1 | 2 | 3 | 4 | 5 |  |
| 608 | During the last 30 days, about how often did you feel that everything was an effort? | 1 | 2 | 3 | 4 | 5 |  |
| 609 | During the last 30 days, about how often did you feel so sad that nothing could cheer you up? | 1 | 2 | 3 | 4 | 5 |  |
| 610 | During the last 30 days, about how often did you feel worthless? | 1 | 2 | 3 | 4 | 5 |  |

**Thank you for your time and responses**
