# Supplementary material for: Anticipated stigma and associated factors among chronic illness patients in Amhara Region Referral Hospitals, Ethiopia: A multicenter cross-sectional study
Source: PLoS One. 2022 Sep 15;17(9):e0273734. doi: 10.1371/journal.pone.0273734 (PMC9477324; doi:10.1371/journal.pone.0273734)
Supplement: Attachment — Submitted filename: Manuscript comments.docx [file pone.0273734.s003.docx]

Manuscript comments

When I review this paper, I have found some strength and some comments to be improved. The paper is well stated and the title is very crucial, very important and timely searched particularly in developing country. The model selection for analysis was appropriate for such type of continuous outcome variable. The result and discussion were described properly. However, I have some comments to be improved the manuscript which is listed below.

1. **In abstract**: methods part better to put first “A simple random sampling technique was used to select the three Referral Hospitals in the region and study subjects.” then “Data were collected using a pre-tested interview based questionnaire.”
2. **In introduction:** better to incorporate about definition of chronic illness and put the definition of stigma (from line 68-72) is in the first paragraph.
3. **In materials and methods part:** The information which is written from line 120 to 122 should be updated. In data collection procedure part in line 203 (each with an MSc –holder **or** higher) needs edition because the data was already collected. From where the pilot studies was done? Should be mention.
